# Theoretical Modeling and Inverse Analysis of Thermal Conductivity of Skeletons in SiO_2_ Nano-Insulation Materials

**DOI:** 10.3390/nano9070934

**Published:** 2019-06-28

**Authors:** Xiao-Chen Zhang, Xin-Lin Xia, Dong-Hui Li, Chuang Sun

**Affiliations:** 1 School of Energy Science and Engineering, Harbin Institute of Technology, Harbin 150001, China; 2 Science and Technology on Space Physics Laboratory, Beijing 100076, China

**Keywords:** nano-insulation materials, thermal conductivity, simulation, identification

## Abstract

With the developments in high-performance nano-insulation material technology, theoretical studies on the heat transfer mechanisms in these materials have been conducted. However, the conductivity of nanometer-sized skeletons is still unclear. It is necessary to clarify the thermal conductivity of nanometer-sized solid skeletons in order to better understand the heat transfer mechanisms in nano-insulation materials. In the present study, a theoretical model for the thermal conductivity of nanometer-sized skeletons in nano-insulation materials is presented based upon the meso-structure of the material and the equation of phonon transfer. The size effect in thermal conductivity of the nanometer-sized particles is studied numerically, and the thermal conductivity is theoretically obtained. At the same time, a reverse method is established for the thermal conductivity of nanometer-sized particles based on the method of particle swarm optimization (PSO). The skeleton thermal conductivity for a specific nano-insulation material with a density of 110 kg/m^3^ and porosity of 0.94 is identified based upon experimental data from literature. Comparison results show that the theoretical conductivity of nanometer-sized skeletons and the identified results give the values of 0.145 and 0.124 W/(m K), respectively, clearly revealing obvious an size effect in the thermal conductivity of nanometer-sized skeletons.

## 1. Introduction

Nano-insulation materials with nanometer-sized pores and solid skeletons have many advantages, such as high specific area, lower thermal conductivity, and light weight, and have found wide applications in many areas such as aerospace applications [1,2] and high performance thermal building insulation [3]. The size of the pores in the materials is usually less than the mean free path (MFP) of air molecules under standard atmospheric conditions; thus, the gas molecule movement is restricted by solid skeletons, leading to a size effect in gas conductivity that decreases with decreasing pore size [4,5]. Similarly, the size effect of thermal conductivity exists in the nanometer-sized solid skeletons that are composed of nano-particles whose diameter is comparable to phonon MFP, leading to a lower thermal conductivity in nanometer-sized skeletons than in solid materials [6].

Experimental and theoretical studies on the thermal performance of nano-insulation materials have been conducted since the 1990s. Theoretical models were established for gas conduction [4,5] in nanometer-sized pores, solid skeleton, and gas coupled conduction [6,7,8] in nanometer scales. Although the thermal performance of nano-insulation materials has been studied, research on the conduction properties of particles in these materials is limited. Han et al. [8] simulated the thermal conductivity of aerogel using the Lattice Boltzmann method and proposed a model for solid and gas combined conduction in which the non-uniform distribution of solid was taken into account. Li et al [9] established a two-dimensional (2D) computational model based upon a phonon radiative transfer equation for phonon transport in a 2D rectangular SiO_2_ nanowire, and numerically studied the size effects in the longitudinal and transverse conductivity. Results show that an obvious size effect in the conductivity exists in the 2D SiO_2_ nanowire, and the thermal conductivity of the wire with a diameter from 2 to 4 nm is 15% less than that of the bulk material. Han et al. [10] studied the phonon transport characteristics in a SiO_2_ nano-membrane using the Lattice Boltzmann method; the thermal conductivity across the membrane was studied, and the size effect of the membrane thermal conductivity was obtained when the Knudson number (Kn) was larger than 0.01. Roberts et al. [11] numerically studied the effective thermal conductivity of films using a molecular dynamics simulation. Smith et al. [12] analyzed the effect of sub-continuum heat transport through a nanoporous silica layer using the Lattice Boltzmann method (LBM).

Although studies on the size effect of thermal conductivity in nano SiO_2_ were conducted, the conduction property of nanoparticles and skeletons in nano-insulation materials remains unclear because the structure of nanoparticles and skeletons were not properly considered in previous studies. It is well known that a size effect exists in the thermal conductivity of nanometer-sized skeletons. However, the skeleton conductivity data from different studies is quite different. Moreover, to the knowledge of the authors, no experimental data on the nanometer-sized skeleton conductivity of nano-insulation materials exists in open literatures, and it is difficult to test theoretical thermal conductivity results of nanometer-sized skeletons in nano-insulation materials. Therefore, it is necessary to clarify the thermal conductivity of nanometer-sized skeletons to gain a better understanding of the heat transfer mechanisms in these materials.

In this paper, nanometer-sized skeleton conductivity was studied using a theoretical method based upon the mesostructure of the material and a reversed method simultaneously. Size effects in the thermal conductivity of the nanometer-sized particles in the materials were studied numerically. At the same time, a reversed identification method for the thermal conductivity of the nanometer-sized particles was established by the method of particle swarm optimization (PSO) based on experimental data of equivalent thermal conductivity. The skeleton thermal conductivity and specific area for a nano-insulation material were simultaneously identified. The theoretical value of thermal conductivity of nanometer-sized skeletons and the identified results were compared.

## 2. Theoretical Simulation of Thermal Conductivity of Nanometer-Sized Skeletons in Nano-Insulation Materials

### 2.1. Computational Models

Nanometer-sized skeletons in SiO_2_ nano-insulation materials are composed of secondary particles with a diameter from 2 to 5 nm [13]. The secondary particles themselves have insight structure; they have pores, and the porosity of these particles does not vary noticeably, with an average value of approximately 0.5. The secondary particles are composed of the primary particles whose diameters are less than 1 nm and change less with the parameters in production processes.

In present study, the phonon transfer process in primary particles of SiO_2_ nano-insulation materials is described by the phonon radiative transport equation as follows [14]:

(1) $$\frac{1}{v}\frac{\partial I}{\partial t}+\vec{\Omega }\nabla I=\frac{I_{}^{0}-I}{\Lambda }$$

where *I* is the phonon radiation intensity, *v* the velocity of sound, $\vec{\Omega }$ the direction vector, Λ the phonon MFP, and *I*^0^ the phonon radiation intensity under the state of equilibrium. With Planck distribution, the radiation intensity *I*^0^ is

(2) $$I_{}^{0}=\frac{\sigma T^{4}}{\pi }$$

where *T* is the absolute temperature. The phonon Stefan-Boltzmann constant σ is as follows [15]:

(3) $$\sigma =\pi^{2}k_{B}^{4}/40\hbar^{3}v^{2}$$

where *k_B_* is Boltzmann constant, ℏ=h/π, and *h* = 6.626 × 10^−34^ J s is the Planck constant.

**Figure 1:**
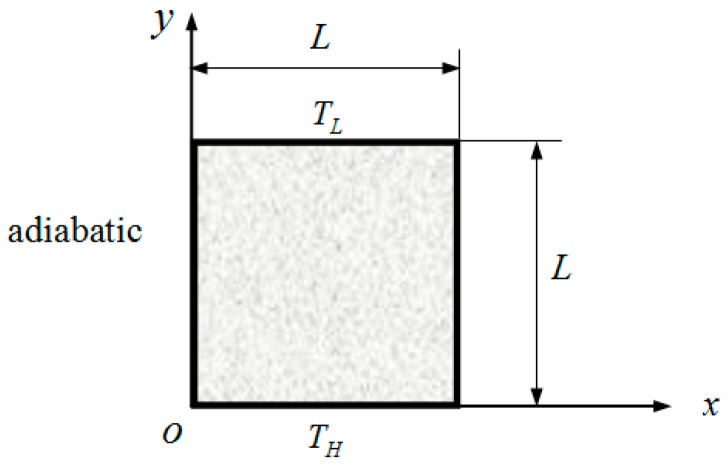
Cross-section of a primary particle chain.

The primary particles are tiny, with a diameter of approximately 1 nm. Although a spherical shape maybe a more reasonable approximation for these particles, in such a scale, which is comparable to the lattice constant, it is hard to consider the shape of primary particles to be ideally spherical. In addition, the primary particles are interconnected, and the contact area is difficult to determine. In the present study, one of the main purposes was to determine the boundary influence on the particle conductivity, with the particle boundary size being the most important factor. To simplify the numerical solving process while maintaining a reasonable precision, the primary particles were assumed to be connected one by one, forming a two-dimensional nanowire with a square cross section as shown in Figure 1.

The temperature boundary condition is adopted on two opposite boundaries [16]:

(4) $${T|}_{y=0}=T_{H}$$

(5) $${T|}_{y=L}=T_{L}$$

where *T_H_*, *T_L_* are boundary temperatures, and the adiabatic condition is adopted on the other two boundaries.

The phonon radiation intensity is derived by solving the equation of phonon radiative transport under steady state by the method of discrete ordinate. The temperature boundary condition can be considered as a blackbody boundary in phonon stimulation. Based upon the obtained phonon radiation intensity, the heat flux density can be calculated as

(6) $$q=\int_{\Omega =4\pi }I\cos\theta d\Omega$$

where Ω is the solid angle. The thermal conductivity of the nanowire composed by the primary particles can be derived from Fourier law:

(7) $$\lambda_{p}=\frac{q}{\left|\nabla T\right|}$$

where |∇T| is the module of the temperature gradient.

### 2.2. Phonon Mean Free Path

In the present study, the concern was the size effect in the thermal conductivity of primary particles in nano-insulation materials; thus, it is reasonable to assume that the primary particles and the bulk material are similar in terms of lattice defects and scattering by phonons. The difference in the phonon MFP between the primary particles and the bulk material is that boundary scattering exists in the primary particles, while in the bulk material boundary scattering does not exist.

By Matthiessen’s law [17], the phonon MFP in primary particles can be determined as

(8) $$\frac{1}{\Lambda }=\frac{1}{\Lambda_{bulk}}+\frac{1}{\Lambda_{b}}$$

where Λ*_bulk_* is the phonon MFP in bulk material, and Λ*_b_* is the phonon MFP caused by boundary scattering. For a two-dimensional rectangular geometry, Λ*_b_* is determined as

(9) $$\frac{1}{\Lambda_{b}}=\frac{1}{B}\left(\frac{1}{w}+\frac{1}{b}\right)$$

where *b* and *w* are the length and width of the rectangle, respectively. For blackbody boundaries, *B* = 0.75 [17].

### 2.3. Simulation of Thermal Conductivity of SiO_2_ Nanometer-Sized Skeletons

The size effect in the thermal conductivity of primary particles in SiO_2_ nano-insulation materials was simulated numerically at room temperature. The material is considered as gray medium. The phonon MFP in the bulk material is Λ*_bulk_* = 0.6 nm, with *c_V_* = 1.79 ×10^6^ J/(m^3^ K), and the average velocity is *v* = 4100 m/s [18]. From the above parameters, the theoretical conductivity of the bulk material can be calculated from phonon kinematic theory as follows:

(10) $$\lambda_{bulk}=\frac{1}{3}c_{V}v\Lambda_{bulk}$$

which has a theoretical value of 1.47 W/(m K).

Under the boundary conditions with *T_H_* = 301 K and *T_L_* = 299 K, the phonon radiation intensity is obtained by numerically solving Equation (1). According to Equations (6) and (7), the thermal conductivity of the particle can be calculated.

**Figure 2:**
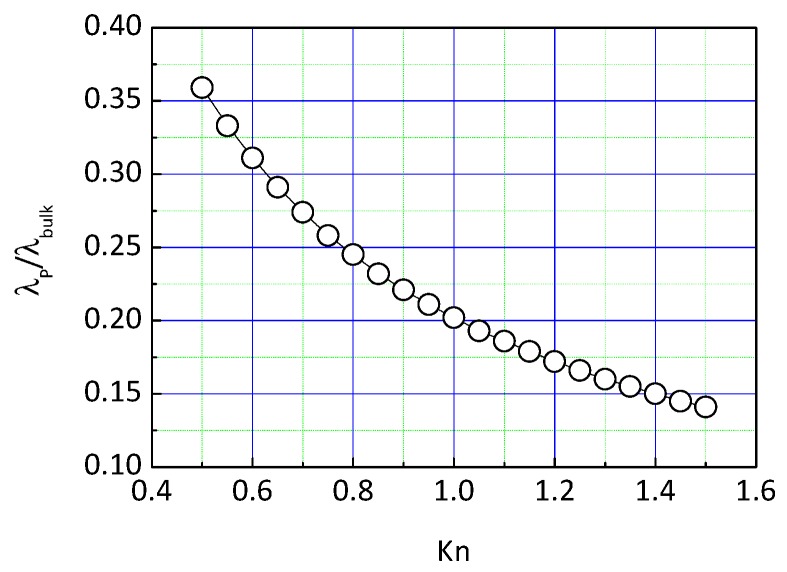
Knudson number (*Kn*) influence on thermal conductivity of primary particle chains.

Figure 2 shows the variation of thermal conductivity of the particle with the Knudson number, which is defined as the ratio of the MFP to the characteristic length *L*.

(11) $$Kn=\frac{\Lambda_{bulk}}{L}$$

The diameter of primary particles in SiO_2_ nano-insulation materials is less than 1 nm, which is comparable to the phonon MFP. It can be seen from the figure that the thermal conductivity of primary particles is clearly less than that of the bulk material.

By linear fitting, a formula between the thermal conductivity of SiO_2_ primary particle chains and the *Kn* is obtained as follows:

(12) $$\frac{\lambda_{P}}{\lambda_{bulk}}={\left(0.637+4.31Kn\right)}^{-1},\left(0.5\leq Kn\leq 1.5\right)$$

**Figure 3:**
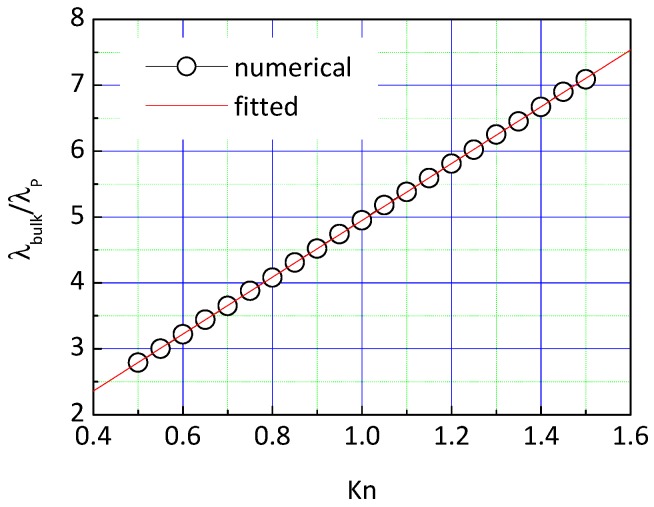
Comparison between fitted conductivity of primary particle chains and numerical data.

Figure 3 presents the comparison between the fitted conductivity and the numerical results. It can be seen that the fitted results and the numerical data are well matched.

Studies on the influence of manufacturing parameters on the diameter of primary particles revealed that the diameter of primary particles in SiO_2_ nano-insulation materials is less than 1 nm, and the diameter varies less obviously with the manufacturing process [19], with an average value of 0.9 nm approximately.

In present study, a two-dimensional computational model with a square cross-section was established for a primary particle chain. The length of the square in the established model can be determined according to the criteria that the spherical primary particle with diameter *d*_p_ has the same surface area as a cube with the side length *L*. Consequently, the relation between the model parameter L and the primary particle diameter *d*_p_ can be obtained as follows:

(13) $$L=\sqrt{\frac{\pi }{6}}d_{p}$$

For primary particles with an average diameter *d*_p_ of 0.9nm, the model parameter *L* = 0.65 nm and *Kn* = 0.923. From Equation (12), the ratio of the thermal conductivity of the primary particles to that of the bulk material can be calculated and has a value of 0.217 W/(m K). The thermal conductivity of SiO_2_ bulk material has an experimental value of 1.34 W/(m K) [17], and the thermal conductivity of primary particles is

*λ_p_* = 0.291 W/(m K) (14)

The secondary particles are composed of primary particles and have pores with a porosity of approximately 0.5 [13,19]. Thus, the thermal conductivity of SiO_2_ secondary particles is

*λs* = 0.5*λp* = 0.145 W/(m K) (15)

which is the thermal conductivity of the skeletons in SiO_2_ nano-insulation materials.

## 3. Identification of Thermal Conductivity of Skeletons in Nano-Insulation Materials

### 3.1. Nanometer-Sized Skeleton and Gas Conduction Model

**Figure 4:**
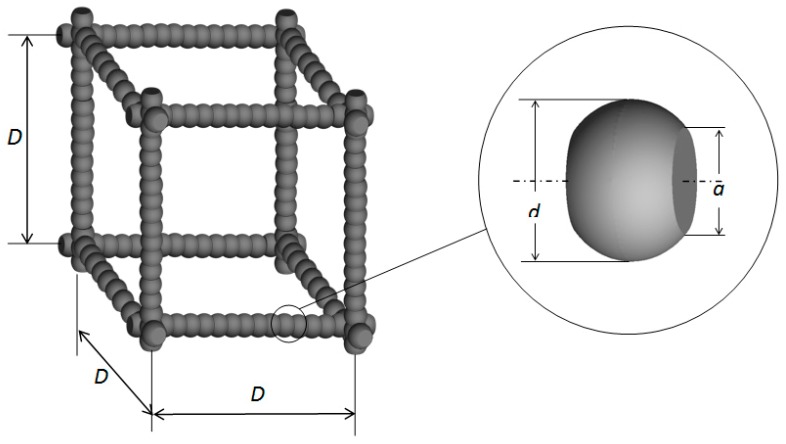
Diagram of cubic array model for coupled conduction in nano-skeletons and gas.

Cubic array models for the coupled conduction of nanometer-sized skeletons and gas in nano-insulation materials are commonly used in most studies. Figure 4 shows the diagram of the cubic sphere array model where the nano-skeletons are composed of nanometer-sized uniform spheres with a diameter *d*, and the diameter of the contact area between adjoining nanometer-sized spheres is *a*. The side length of the cube is *D*, corresponding to the mean diameter of pores in aerogels. The other coupled conduction model commonly used is the cubic cylinder array model, which is similar to the cubic sphere array model mentioned above. In the cubic cylinder array model the shape of the nano-skeletons is a cylinder with a diameter *d*.

Zeng [6] indicated that the difference in the effective thermal conductivity results obtained from the cubic cylinder and the cubic sphere array models was not obvious. Thus, the cubic cylinder array model was used in this study.

The effective thermal conductivity for the nanometer-sized coupled conduction in the cubic cylinder array model is described as

(16) $$\lambda_{c}=\frac{\pi }{4}{\left(\frac{d}{D}\right)}^{2}\lambda_{s}^{}+{\left(1-\frac{d}{D}\right)}^{2}\lambda_{g}^{}+\frac{\pi -B_{2}}{B_{1}}\frac{d}{D}\left(1-\frac{d}{D}\right)\lambda_{g}^{}$$

The model parameters of *B*_1_, *B*_2_ are defined as

(17) $$B_{1}=\left(\frac{\lambda_{g}^{}}{\lambda_{s}^{}}-1\right)\frac{d}{D}$$

(18) $$B_{2}=\frac{4}{\sqrt{1-B_{1}^{2}}}\tan^{-1}\left(\sqrt{\frac{1-B_{1}}{1+B_{1}}}\right)$$

In the cubic cylinder array model, the diameter of the nanometer-sized skeleton *d* and the mean diameter *D* of pores can be determined from the parameters of porosity Π and specific area *S* of the insulation material as follows:

(19) $$\Pi =\sqrt{2}{\left(\frac{d}{D}\right)}^{3}-\frac{3\pi }{4}{\left(\frac{d}{D}\right)}^{2}+1$$

(20) $$S=\frac{3\pi dD-6\sqrt{2}d^{2}}{D^{3}\rho }$$

The nano-insulation material of pure SiO_2_ aerogel is not effective in attenuating radiation heat transfer [20], and the insulation material applied in engineering is generally opacified [21,22]. Consequently, the conduction transfer process in the material is coupled with radiation heat transfer. In the present study, the carbon opacified SiO_2_ aerogel was considered and its spectral absorption coefficient is shown in Reference [22]. The spectral absorption coefficient of the opacified aerogel is large in most of the spectral range. It is reasonable to consider the insulation of the opacified aerogel to be optically thick, and Rosseland averaged approximation can be used to obtain its full-spectrum averaged absorption coefficient *k_a,R_*.

(21) $$k_{a,R}=\int_{0}^{\infty }\frac{1}{k_{a\lambda }}\frac{dE_{b\lambda }}{dE_{b}}d\lambda$$

where *k_aλ_* is the spectral absorption coefficient of nano-insulation material, *E_bλ_* is the spectral emission power of a black body, *E_b_* is the emission power of a black body, and *λ* is its wavelength.

**Figure 5:**
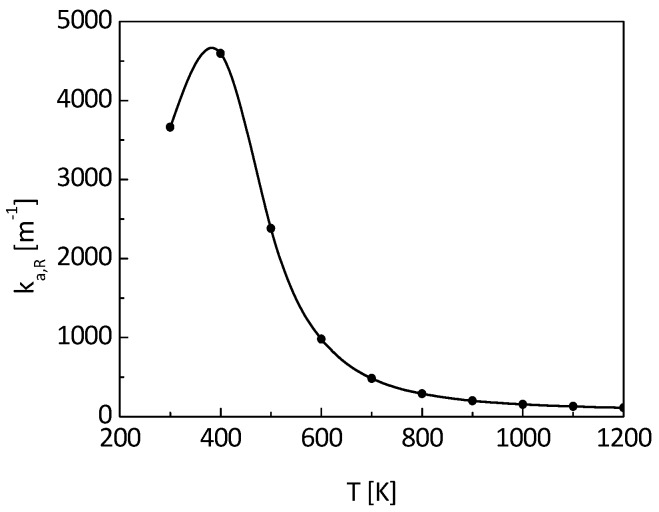
Rosseland averaged absorption coefficient of the aerogel.

Figure 5 shows the Rosseland averaged absorption coefficient *k_a,R_* of the carbon opacified aerogel under various temperatures, which is about 3663 m^−1^ at room temperature.

Under optically thick approximation, the radiative equivalent conductivity is

(22) $$\lambda_{r}=\frac{16n^{2}\sigma T^{3}}{3k_{a,R}}$$

where *n* is the refractive index of the insulation material, *T* is the absolute temperature, and *σ* = 5.67 × 10^−8^ W/(m^2^ K^4^) is the Stefan Bolzmann constant. The refractive index of aerogel can be calculated from the Clausius-Mosotti formula as

(23) n=1+0.2ρ

where *ρ* is the density of the aerogel in g/cm^3^. In this study, the density of the aerogel is 110 kg/m^3^, and its refractive index is 1.02. It can be obtained from Equation (22) that the radiative equivalent conductivity of the considered aerogel at room temperature is about 2.2 × 10^−3^ W/(m K).

Considering both heat conduction and radiation transfer in the aerogel, its equivalent thermal conductivity is

(24) $$\lambda_{eq}=\lambda_{c}+\lambda_{r}$$

Therefore, the effective conductivity of nanometer-sized skeleton and gas in the aerogel is:

(25) $$\lambda_{c}=\lambda_{eq}-\lambda_{r}$$

Reference [6] shows the measured equivalent thermal conductivity under various atmospheric pressures. From Equation (25), the experimental data of effective conductivity of the nano-insulation material can be deduced.

**Figure 6:**
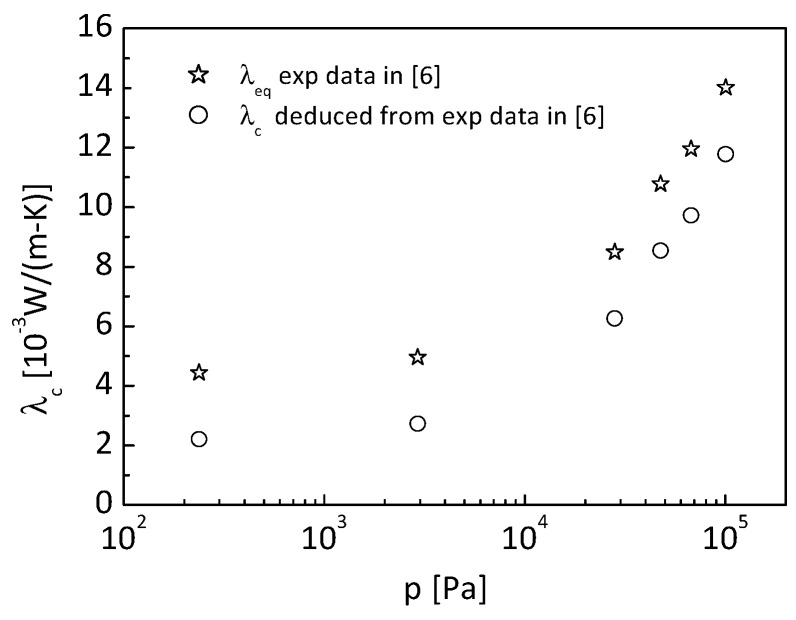
Experimental effective conductivity.

Figure 6 shows the deduced experimental effective conductivity for the specific SiO_2_ aerogel with density of 110 kg/m^3^, specific area of 7.976 × 10^5^ m^2^/kg, and porosity of 0.94 in Reference [6].

It needs to be clarified that the thermal conductivity given in Figure 6 contains the contribution from the conduction in nanometer-sized solid skeletons, in pores, and through the adulteration particles of carbon black. The mass content of carbon black in the SiO_2_ aerogel is 3.25% [6]. It can be easily derived that the carbon black volume content is 0.176%, which is very low. Consequently, the influence of carbon black on thermal conductivity can be neglected.

### 3.2. Identification Method for Thermal Conductivity of Nano-Skeletons in Aerogels

The particle swarm optimization (PSO) method combined with the coupled conduction model of the cubic cylinder model was used to identify the nanometer-sized skeleton conductivity.

**Figure 7:**
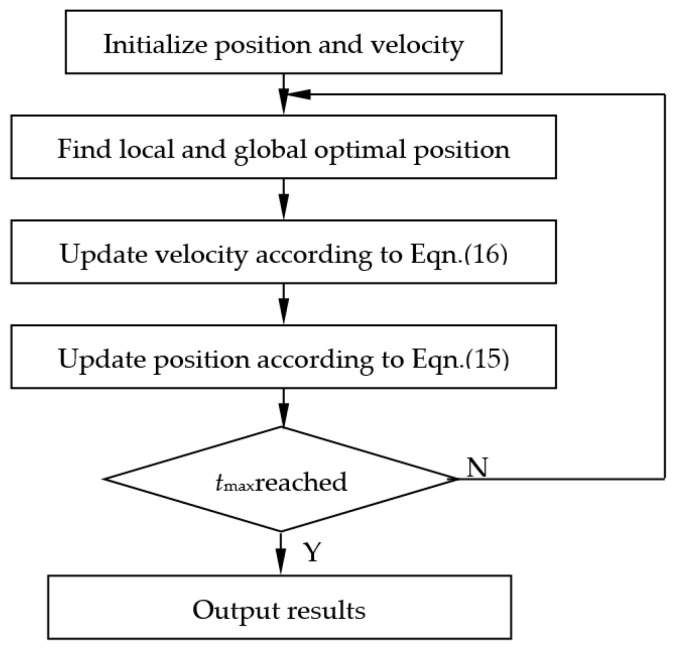
Flowchart of particle swarm optimization (PSO) method.

The PSO method is an intelligent algorithm based upon a searching model of velocity-position. The solution of the optimization problem is treated as a particle swarm flying in a searching space, with each particle having its specific velocity, which can be adjusted dynamically by its experience and position. For a swarm composed of *m* particles in a *d*-dimensional space, the position of the particle labeled *i* in the swarm is $X_{i}=\left(x_{i1},x_{i2},\ldots ,x_{id}\right)$, its velocity is $V_{i}=\left(v_{i1},v_{i2},\ldots ,v_{id}\right)$, and its optimal position (individual optimal adaptability) is $P_{best}=\left(P_{1},P_{2},\ldots ,P_{d}\right)$. The global optimum position among all particles is denoted as $G_{best}=\left(G_{1},G_{2},\ldots ,G_{d}\right)$. The position and velocity of each particle in the next iteration are determined by the following equations:

(26) $$V_{ij}\left(t+1\right)=wV_{ij}\left(t\right)+C_{1}R_{1}\left[P_{j}\left(t\right)-x_{ij}\left(t\right)\right]+C_{2}R_{2}\left[G_{j}\left(t\right)-x_{ij}\left(t\right)\right],\left(j=1,2,\ldots ,d\right)$$

(27) $$x_{ij}\left(t+1\right)=x_{ij}\left(t\right)+V_{ij}\left(t+1\right),\left(j=1,2,\ldots ,d\right)$$

where *C*_1_, *C*_2_, usually equal to 2, are known as the learning factors, which adjust the maximum step towards the global and local optimal particle positions; *w* is the inertial weight.

(28) $$w=w_{\max}-\frac{t}{t_{\max}}\left(w_{\max}-w_{\min}\right)$$

where *w*_max_ is the maximum inertial weight, *w*_min_ is the minimum inertial weight, *t* is the iteration number and *t*_max_ is the maximum iteration number. In present study *w*_max_ and *w*_min_ are set to be 0.9 and 0.4 respectively. The optimization process iterates based upon Equations (26) and (27) until the maximum iteration number is reached or the solution precision is satisfied. Figure 7 shows the flow chart of the PSO method.

### 3.3. Identification of Thermal Conductivity of Nanometer-Sized Solid Skeletons

The thermal conductivity of the nanometer-sized solid skeletons in the carbon opacified aerogel with a density of 110 kg/m^3^ and porosity of 0.94 was identified using the method of PSO under the condition where the specific area of 7.976 × 10^5^ m^2^/kg is regarded as a given parameter. The objective function is defined as

(29) $$\min f\left(\lambda_{s}\right)=\sqrt{\frac{1}{N}\sum_{i=1}^{N}{\left[\lambda_{c}^{\exp}\left(p_{i}\right)-\lambda_{c}^{num}\left(\lambda_{s},p_{i}\right)\right]}^{2}}$$

where $\lambda_{c}^{\exp}\left(p_{i}\right)$ is the measured effective conductivity of the opacified aerogel under the atmospheric pressure of *p_i_*, and $\lambda_{c}^{num}\left(\lambda_{s},p_{i}\right)$ are the theoretical results obtained from the cubic cylinder array model. The bulk material of the aerogel considered is SiO_2_, with a thermal conductivity of about 1.34 W/(m K); thus, the thermal conductivity of the nanometer-sized skeleton is constrained by a range of 0.01–1.34 W/(m K). The experimental effective thermal conductivity of the aerogel under various pressures is shown in Figure 6. The experimental data on effective conductivity is assumed to be free of stochastic errors in the identification process. The particle number is set to be 20 with the maximum generation number of 1000.

**Figure 8:**
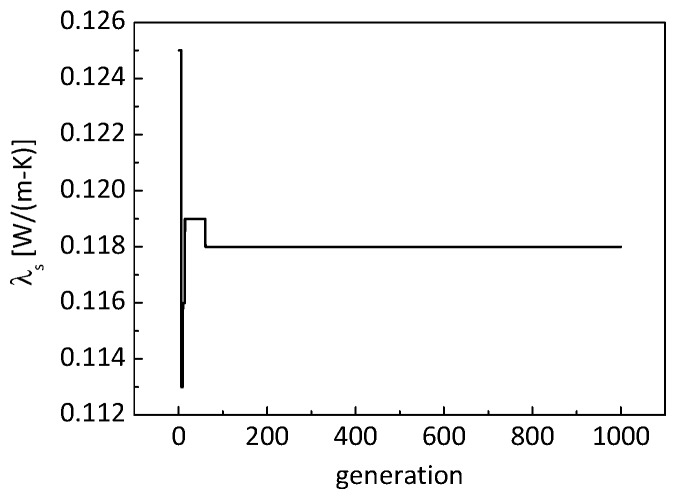
Evolution of identified thermal conductivity of nanometer-sized solid skeletons.

Figure 8 shows the evolution of the identified thermal conductivity of the nanometer-sized solid skeletons. It can be seen that the identified conductivity converged in less than 100 generations with 20 particles, and the identified conductivity of nanometer-sized skeletons was about 0.118 W/(m K).

**Figure 9:**
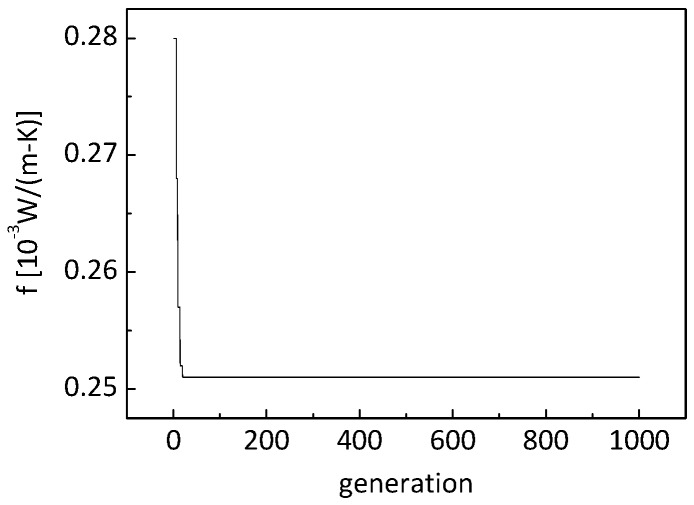
Evolution of the objective function.

Figure 9 shows the evolution of the objective function. It can be seen that the converged value of the objective function is about 2.51 × 10^−4^ W/(m K).

**Figure 10:**
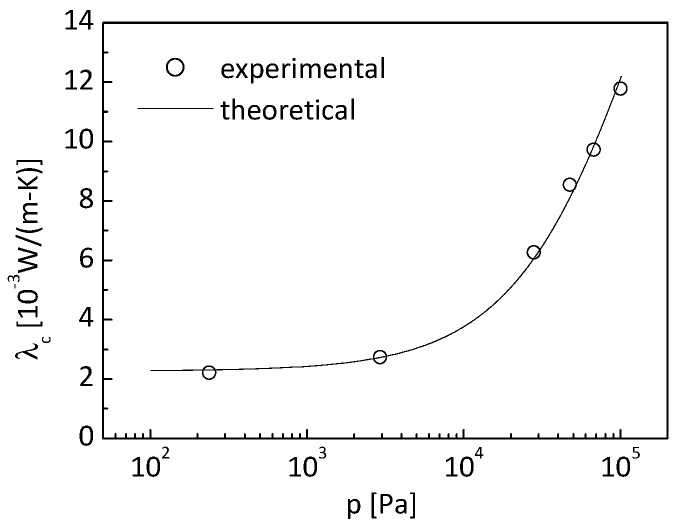
Effective thermal conductivity obtained from the theoretical model based upon the identified skeleton conductivity.

Figure 10 shows the effective thermal conductivity obtained from the cubic cylinder array model based upon the identified skeleton conductivity. It can be observed that the theoretical results match the deduced experimental data very well.

Table 1 shows the influence of the stochastic error *e* in the measured effective conductivity on the identified conductivity of nanometer-sized skeletons. It can be observed that the stochastic error *e* of less than 20% in the measured data has little influence on the identified nanometer-sized skeleton conductivity. The deviation in the identified conductivity is less than 0.2%.

### 3.4. Identification of Thermal Conductivity of Nanometer-Sized Solid Skeletons and Specific Area

The thermal conductivity of the nanometer-sized solid skeletons and specific area of the aerogel with a density of 110 kg/m^3^ and porosity of 0.94 were identified simultaneously. The objective function is defined as

(30) $$\min f\left(\lambda_{s},S\right)=\sqrt{\frac{1}{N}\sum_{i=1}^{N}{\left[\lambda_{c}^{\exp}\left(p_{i}\right)-\lambda_{c}^{num}\left(\lambda_{s},S,p_{i}\right)\right]}^{2}}$$

where $\lambda_{c}^{num}\left(\lambda_{s},S,p_{i}\right)$ is the effective conductivity obtained from the theoretical model of the cubic cylinder array based upon the specific area *S*, thermal conductivity *λ_s_* of the nanometer-sized solid skeletons, and the atmospheric pressure *p_i_*. It is assumed that the stochastic error does not exist in the measured data.

**Figure 11:**
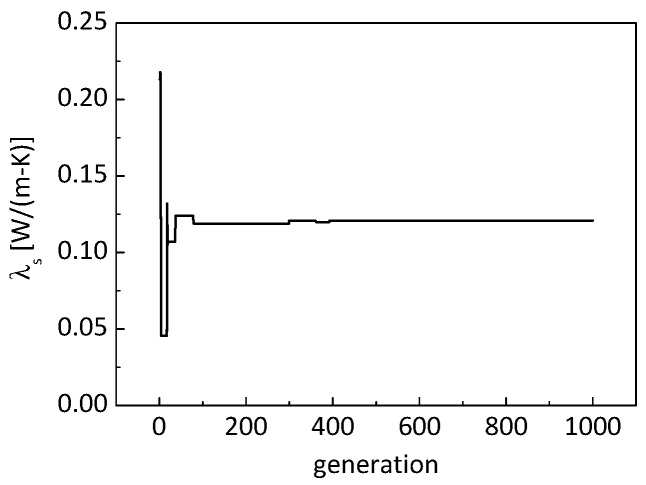
Evolution of the identified thermal conductivity of the nanometer-sized solid skeletons.

**Figure 12:**
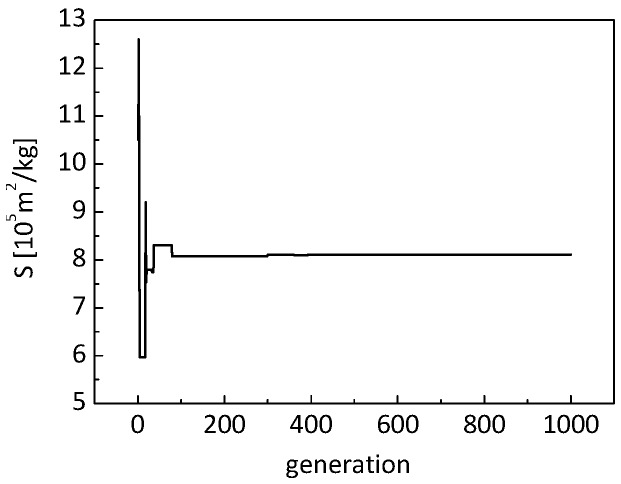
Evolution of the identified specific area.

Figure 11 and Figure 12 show the evolution of the identified thermal conductivity of the nanometer-sized solid skeletons and the identified specific area. It can be observed that the identified conductivity is about 0.1206 W/(m K), and the identified specific area is 8.11 × 10^5^ m^2^/kg. The identified specific area of the material matches its measured value of 7.976 × 10^5^ m^2^/kg very well, with a relative error of less than 2%. Consequently, it is reasonably deduced that the identified conductivity of the nanometer-sized solid skeletons is reliable. In addition, the identified conductivity of 0.1206 W/(m K) matches the identified value of 0.118 W/(m K) in the previous section.

**Figure 13:**
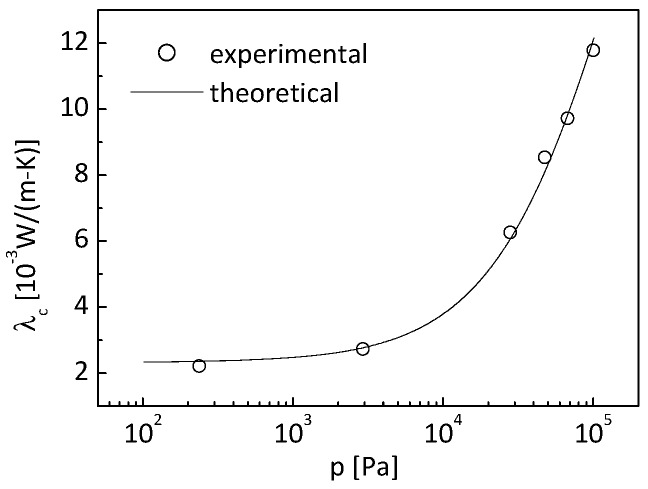
Effective thermal conductivity obtained from theoretical model based upon the identified skeleton conductivity and specific area.

Figure 13 shows the effective thermal conductivity obtained from the cubic cylinder array model based upon the identified skeleton conductivity and specific area, and its comparison with the experimental values. It can be observed that the theoretical results match the deduced experimental data very well as in the previous section.

Table 2 and Table 3 show the influence of the stochastic error in the measured data on the identified results. It can be observed that the identification precision of the nanometer-sized skeleton conductivity and specific area decreases with the increase in measurement uncertainty. The stochastic error in the measured data has a small influence on the identified results. With an error of less than 20% in the measured effective conductivity, the identified thermal conductivity of nanometer-sized skeletons has a relative error of less than 6.5%, and the identified specific area has a relative error of less than 4.5%.

## 4. Comparison of Identified Thermal Conductivity of Skeletons in Nano-Insulation Materials with Theoretical Results

Table 4 shows the comparison between the theoretical and identified values of the thermal conductivity of nanometer-sized skeletons. It can be seen that the deviation of the identified results from the theoretical results is not substantial. The deviation of the theoretical results from those identified is less than 20%.

## 5. Conclusions

The presented theoretical model based upon the mesostructure of nano-insulation materials and the equation of phonon radiative transfer is feasible. The method to identify the thermal conductivity of nanometer-sized solid skeletons, which is established using PSO and a cubic cylinder array conduction model, was tested to be reliable.

For a specific nano-insulation material with a density of 110 kg/m^3^ and porosity of 0.94, the theoretical analysis yielded a thermal conductivity of the nanometer-sized skeleton in the aerogel of approximately 0.145 W/(m K), which was much less than the bulk material conductivity. The identified thermal conductivity of nanometer-sized solid skeletons was 0.1206 W/(m K). Theoretical and identified results of the thermal conductivity of nanometer-sized skeletons are well matched, clearly revealing an obvious size effect in the thermal conductivity of nanometer-sized skeletons.

## Figures and Tables

**Table 1:** Influence of stochastic error in the measured data on the identified conductivity of nanometer-sized skeleton.

*e* (%)	0	5	10	15	20
*λ_s_* (W/(m K))	0.1183	0.1184	0.1184	0.1185	0.1185

**Table 2:** Influence of stochastic error *e* in the measured data on the identified conductivity of nanometer-sized skeleton and specific area.

*e* (%)	0	5	10	15	20
*λ_s_* (W/(m K))	0.1206	0.1225	0.1244	0.1264	0.1283
*S* (× 10^5^ m^2^/kg)	8.1107	8.1998	8.2906	8.3831	8.4774

**Table 3:** Influence of stochastic error *e* in the measured data on the relative error of the identified conductivity of nanometer-sized skeleton and specific area.

*e* (%)	5	10	15	20
$e_{\lambda_{s}}$(%)	1.6	3.2	4.8	6.4
$e_{s}$(%)	1.1	2.2	3.4	4.5

**Table 4:** Comparison between theoretical and identified values of thermal conductivity of nanometer-sized skeletons.

*e* (%)	Single Parameter Identification		Double Parameter Identification		Theory
	*λ_s_* (W/(m K))	Deviation (%)	*λ_s_* (W/(m K))	Deviation (%)	
0	0.1183	−18.4	0.1206	−16.8	0.145
5	0.1184	−18.3	0.1225	−15.5	
10	0.1184	−18.3	0.1244	−14.2	
15	0.1185	−18.3	0.1264	−12.8	
20	0.1185	−18.3	0.1283	−11.5	

## Nomenclature

*I*	phonon radiation intensity, W/(m^2^ sr)
*v*	velocity of sound, m/s
Λ	phonon MFP, nm
*T_H_, T_L_*	boundary temperature, K
*q*	heat flux density, W/m^2^
*λ_p_* *, λ_s_*	thermal conductivity of primary and secondary particles, W/(m K)
*Kn*	Knudson number
*d_ρ_*	diameter of nanometer-sized spheres, m
*D*	mean diameter of pores in aerogels, m
*λ_c_*	effective thermal conductivity for nanometer-sized coupled conduction, W/(m K)
*λ_g_*	thermal conductivity of gas in pores, W/(m K)
Π	porosity
*S*	specific area, m^2^/kg
*B_1_, B_2_*	parameters defined in Equations (7) and (8)
*ρ*	density of nano-insulation material, kg/m^3^
*k_a,R_*	spectrum averaged absorption coefficient, 1/m
*k_aλ_*	spectral absorption of nano-insulation material, 1/m
*E_bλ_*	spectral emission power of black body, W/(m^2^ μm)
*E_b_*	emission power of black body, W/m^2^
*λ*	Wavelength, μm
*λ_r_*	radiative equivalent conductivity, W/(m K)
*T*	absolute temperature, K
*λ_eq_*	equivalent thermal conductivity of nano-insulation material, W/(m K)
*m*	number of particles in swarm
*X_i_*	position vector of a particle labeled *i* in a swarm
*V_i_*	velocity vector of a particle labeled *i* particle in a swarm
*P_best_*	individual optimal adaptability
*G_best_*	global optimum position among all particles
*V_ij_*	*j*th component of velocity vector of a particle labeled *i* in a swarm, (*i* = 1,…,*m*)
*X_ij_*	*j*th component of position vector of a particle labeled *i* in a swarm, (*i* = 1,…,*m*)
*C_1_* *, C_2_*	learning factors
*w*	inertial weight
*t*	number of iteration
